# Supplementary material for: WordCommentsAnalyzer: A windows software tool for qualitative research
Source: F1000Res. 2018 Sep 4;7:536. Originally published 2018 May 3. [Version 2] doi: 10.12688/f1000research.14819.2 (PMC6137411; doi:10.12688/f1000research.14819.2)
Supplement: Supplementary file 3 [file f1000research-7-17614-s0002.tgz › b2a79496-4ada-47c4-9682-a99ce867836d.zip!/Eight Iranian Health Journals/Journal of Knowledge and Health_8.docx]

© Journal of Knowledge and Health. This work is licensed under CC BY 4.0 (https://creativecommons.org/licenses/by/4.0/).

Volume & Issue: دوره 11، شماره 2: 1395

Title: رابطه علی ویژگی‌های شخصیتی و رشد پس از سانحه، با میانجی‌گری تنظیم شناختی- هیجانی در بیماران مبتلا‌به هپاتیت B

http://knh.shmu.ac.ir/index.php/site/article/view/1154

مقدمه: رشد پس از سانحه بعد از سوانحی مانند بيماري‌های مزمن در برخی از افراد به‌وجود می‌آید که عوامل مختلف روانشناختی می‌توانند در شکل گیری آن تأثیر داشته باشند. لذا از آنجا که رشد پس از سانحه در بیماری هپاتیت B تاکنون مورد مطالعه قرار نگرفته است، این پژوهش با هدف بررسی رابطه علی ویژگی‌های شخصیتی و رشد پس از سانحه با میانجی‌گری تنظیم شناختی- هیجانی در بیماران مبتلا‌به هپاتیت B تدوین گردید. مواد و روش‌ها: جامعه آماری این پژوهش شامل بیماران مبتلا‌به هپاتیت B در مرکز تحقیقات هپاتیت بیمارستان نمازی شیراز بود. 165 بیمار مبتلا‌به هپاتیت B (95 زن و 70 مرد) به‌طور تصادفی انتخاب شدند و از پرسشنامه‌های شخصیتی کلونینجر، پرسشنامه تنظیم شناختی- هیجانی گارنفسکی و پرسشنامه رشد پس از سانحه تدسچی استفاده شد. جهت تحلیل داده‌ها از نرم‌افزار SPSS و AMOS و تحلیل مسیر استفاده شد. نتایج: نتایج نشان داد که ویژگی‌های شخصیتی وابستگی به پاداش، تعالی‌بخشی خود، خودراهبری، همکاری، پشتکار و تنظیم شناختی- هیجانی اثر مستقیم و معناداری بر رشد پس از سانحه دارند که ضریب Beta مربوط‌به این متغیرها به‌ترتیب شامل 082/0، 074/0، 064/0، 451/0، 081/0 و 380/0 می‌باشد. همچنین نتایج نشان داد که نوجویی، آسیب پرهیزی، وابستگی به پاداش و همکاری از طریق تنظیم شناختی- هیجانی بر رشد پس از سانحه اثر دارد که ضریب  Betaمربوط‌به این متغیرها به‌ترتیب شامل 084/0-، 067/0-، 086/0، 141/0 می‌باشد. نتیجه‌گیری: ویژگی‌های شخصیتی علاوه بر اثر مستقیم بر رشد پس از سانحه، از طریق تنظیم شناختی- هیجانی نیز به‌طور غیرمستقیم بر رشد پس از سانحه تأثیر دارد.

Title: بررسی تأثیر عصاره متانولی برگ، گل و ساقه گیاه دافنه ماکروناتا بر ترمیم زخم پوستی در رت

http://knh.shmu.ac.ir/index.php/site/article/view/1266

مقدمه: دافنه ماکروناتا (Daphne mucronata) دارای خاصیت آنتی‌اکسیدانی و ضدالتهابی بوده و می‌تواند فرآيند ترمیم زخم را تسهیل نماید. هدف از این تحقیق بررسی تأثیر دافنه ماکروناتا بر روی ترمیم زخم پوستی موش صحرایی بالغ می‌باشد. مواد و روش‌ها: در این مطالعه تجربی، 45 سر رت نر مورد استفاده قرار گرفت که به‌طور تصادفی در چهار گروه عصاره متانولی 98% (گل، ساقه، برگ) و شاهد به مدت بیست و دو روز تیمار شدند. براي اين منظور زخمي به‌صورت دایره‌ای به قطر 1سانتی‌متر در ناحیه پوست پشت گردن ایجاد و نمونه‌هایی در روزهای سوم، هفتم، چهاردهم و بیست و دوم بعد از تیمار برای مطالعه میکروسکوپی گرفته شد. توسط نرم‌افزار SPSS نسبت‌به تجزيه و تحليل داده‌ها اقدام و نتایج به‌وسیله آزمون کروسکالوالیس و فریدمن با هم مقایسه شدند. نتايج: از نظر ماکروسکوپی و اندازه‌ سطح زخم در نمونه‌های درمانی (یا عصاره گل، ساقه و برگ) روند ترمیم و بهبودی زخم به‌طور چشمگیری روبه افزایش بود، به‌طوری‌که در چهاردهمین روز ایجاد زخم، بهبودی کامل حاصل گردید و در روز بیست و دوم حتی جای زخم قابل تشخیص نبود. همچنین درصد بهبودی زخم در روز چهاردهم و بیست و دوم بین گروه‌های تیمار با شاهد، اختلاف معنی‌داری مشاهده شد (05/0<P). نتایج هیستوپاتولوژیک نشان دادکه عصاره دافنه (گل، برگ و ساقه) می‌تواند بر ترمیم زخم پوستی مؤثر باشد. نتیجه‌گیری: نتایج نشان دادکه عصاره‌های متانولی تهیه شده از گل، ساقه و برگ گیاه دافنه ماکروناتا در بهبودی و افزایش سرعت ترمیم زخم‌های پوستی مؤثر می‌باشد.

Title: شیوع عفونت هلیکوباکتر پیلوری در زنان باردار ایران- یک مطالعه متاآنالیز

http://knh.shmu.ac.ir/index.php/site/article/view/1255

مقدمه: هلیکوباکترپیلوری، باکتری شناخته شده‌ای است و در ایجاد بیماری‌های گوارشی متعدد نقش دارد. مطالعات متعدد در ایران، شیوع هلیکوباکتر پیلوری در زنان باردار را در محدوده 7/7 تا 80 درصد گزارش کرده‌اند و برآورد کلی از آن وجود ندارد. مطالعه حاضر با هدف برآورد شيوع هلیکوباکتر پیلوری در زنان باردار ایرانی با استفاده از روش متاآناليز انجام شده است. مواد و روش‌ها: با‌توجه به هدف مطالعه، مقالات چاپ شده در مجلات داخلی و خارجی در خصوص برآورد شیوع عفونت هلیکوباکترپیلوری در زنان باردار ایرانی مورد بررسی قرار گرفتند. به‌منظور دستيابي به مستندات مرتبط، بانک‌های اطلاعاتی داخلی و بین‌المللی شامل: Magiran، IranMedex، IranDoc، SID، Medlib، Scopus، Pubmed، Web of Sciences، Medline، ScienceDirect، Cochrane، Embase و همچنین موتور جستجوی Google Scholar بدون محدویت زمانی تا سال 1394 با استفاده از کلید واژه‌های استاندارد مورد جستجو قرار گرفتند. جستجو، استخراج داده و ارزیابی کیفی مقالات توسط دو نفر از پژوهشگران به‌صورت مستقل انجام شد. داده‌ها با استفاده از مدل اثرات تصادفی به‌روش متاآنالیز و نرم‌افزار Stata نسخه 1/11 مورد تجزيه و تحليل آماري قرار گرفتند. نتایج: در این بررسی 22 مطالعه با حجم نمونه 1605 نفر مورد بررسی قرار گرفتند. شیوع هلیکوباکترپیلوری در زنان باردار ایرانی 9/45% (5/52-4/39: 95%) فاصله اطمینان برآورد گردید. کمترین و بیشترین شیوع هلیکوباکترپیلوری در زنان باردار مربوط‌به غرب (2/36%) و جنوب کشور (5/61%) است. نتیجه‌گیری: شیوع هلیکوباکترپیلوری در زنان باردار ایرانی بالا و در حد جمعیت عمومی ایران می‌باشد و باید استراتژی‌های پیشگیرانه جهت کنترل این عفونت انجام شود.

Title: تأثیر تمرینات تناوبی شدید (HIT) بر سطوح پلاسمایی TNF-a، IL-6 و نیمرخ لیپیدی کودکان دارای اضافه وزن

http://knh.shmu.ac.ir/index.php/site/article/view/1181

مقدمه: شیوع چاقی در بین کودکان و نوجوانان در سراسر جهان به‌صورت نگران‌کننده‌ای در حال افزایش است. فعالیت ورزشی منظم می‌تواند در کاهش بافت چربی و بهبود ترکیب بدن نقش داشته باشد. هدف پژوهش حاضر تأثیر تمرینات تناوبی شدید (HIT) بر سطوح پلاسمایی TNF-a، IL-6 و نیمرخ لیپیدی کودکان دارای اضافه وزن بود. مواد و روش‌ها: تحقیق حاضر از نوع نیمه تجربی بود که در سال 1394 در استان تهران انجام شد. در پژوهش حاضر 32 نفر از دانش‌آموزان دارای اضافه وزن 8 تا 11 ساله به دو گروه تجربی (16=n) و کنترل (16=n) تقسیم شدند. تمرینات HIT شامل وهله‌های 30 ثانیه‌ای با حداکثر سرعت و 5/1 دقیقه استراحت به مدت 8 هفته و 3 جلسه تمرین در هفته اجرا شد. تعداد وهله‌ها از 6 وهله در هفته اول به 10 وهله در هفته آخر افزایش یافت. نمونه‌های خونی 48 ساعت پس از آخرین جلسه تمرین از ورید بازوی راست آزمودنی‌ها جمع‌آوری شد. از آزمون تحلیل کواريانس با سطح معنی‌داری (05/0=a) جهت مقايسه ميانگين متغيرها اجرا شد. نتایج: یافته‌های پژوهش حاضر نشان داد که 8 هفته تمرینات HIT مقادیر IL-6 (001/0=P)، TNF-a (001/0=P) و کلسترول (001/0=P) به‌طور معنی‌داری کاهش یافت. از طرف دیگر تغییرات غیر معنی‌داری در مقادیر تری‌گلیسرید (777/0P=)، LDL (071/0P=) و HDL (693/0P=) مشاهده شد.نتیجه‌گیری: یافته‌های پژوهش حاضر نشان داد که هشت هفته تمرین HIT باعث کاهش مقادیرTNF-a و IL-6 می‌شود. لذا انجام تمرینات HIT با رعایت احتیاط‌های لازم در کودکانی که دارای اضافه وزن هستند می‌تواند اثرات مثبتی داشته باشد.

Title: تمايز سلول‌هاي بنیادی مزانشيمي حاصل از بند ناف به سمت سلول‌هاي شبه عصبي با استفاده Activin A و Nicotinamide

http://knh.shmu.ac.ir/index.php/site/article/view/1367

مقدمه: سلول‌های بنیادی مزانشیمی حاصل از بند ناف يكي از بهترين منابع براي درمان‌هاي مبتنی بر سلول هستند. اين سلول‌ها در شرايط کشت تمايزي، مورفولوژی سلول عصبی را نشان مي‌دهند و مارکرهاي عصبی را بيان مي‌كنند. هدف از مطالعه حاضر تمايز سلو‌‌ل‌هاي بنیادی با استفاده از Activin A و Nicotinamide به سمت سلول‌هاي شبه عصبي مي‌باشد. مواد و روش‌ها: بند ناف نزدیک به جفت بريده، نمونه‌هاي کوچک‌تر (2-4 سانتی‌متري) تهيه شد و ژله وارتون جدا گرديد. سلول‌هاي بنيادي از طريق روش اكسپلنت استحصال گرديد و فنوتیپ سطح سلول توسط دستگاه فلوسيتومتري Dako و با نرم‌افزار FlowJo مورد تجزیه و تحلیل قرار گرفت. سپس تمایز اديپوژنيك و استئوژنیک سلول‌ها صورت گرفت. سلول‌ها در شرايط كشت تمايز عصبي قرار داده شد و به مدت 3 روز در محيط كشت RPMI تحت تيمار باActivin  20 μg/ml و 1/0%FBS قرار گرفته و سپس به مدت 7 روز تحت القاي 10 ميلي‌مولار nicotinamide، B27 2%و 1/0%FBS قرار گرفتند. نتایج: 10-7 روز پس از كشت اكسپلنت، سلول‌های بنيادي مزانشيمي دوکی شکل مشاهده شد. ایمونوفنوتیپ سلول‌های بنیادی کشت شده براي ماركرهاي مزانشیمی مانندCD 105 و CD 90 مثبت بود اما براي ماركرهاي خون‌ساز CD34 و CD45 منفي بود.پس از القا در محيط كشت تمايزي اديپوژنيك و استئوژنيك، قطرات چربي و رسوبات کلسیم در سلول‌ها مشاهده شد. سلول‌های شبه عصبي تمايزيافته به شكل سلول‌هاي هرمي شكل با زوائد نوروني ظاهر گرديد. در طي القاي عصبی، زوائد شبه اكسوني و دندريتي در برخي از سلول‌هاي دو قطبي و چند قطبي مشاهده گرديد. همچنين در بين سلول‌ها تماس سلولي مستقيم شبه سيناپسي مشاهده گرديد. نتیجه‌گیری: نتایج مطالعه حاضر نشان داد کهActivin A و nicotinamide باعث القاي تمایز سلول‌های بنیادی مزانشیمی به سمت دودمان عصبي گرديد.

Title: بررسی روایی و پایایی پله با قابلیت تنظیم ارتفاع جهت انجام آزمون پله

http://knh.shmu.ac.ir/index.php/site/article/view/1354

مقدمه: یکی از روش‌های غیرمستقیم اندازه‌گیری بيشترين ظرفيت هوازي، آزمون پله می‌باشد. بیشتر آزمون‌های پله، بر روی پله‌هایی با ارتفاع ثابت طراحی شده‌اند که تفاوت‌های بیومکانیکی ناشی از قد افراد می‌تواند باعث سوگرایی در نتایج شود. مطالعه حاضر با هدف تعیین روایی و پایایی پله‌ای با قابلیت تنظیم ارتفاع جهت آزمون پله انجام شده است. مواد و روش‌ها: حداقل و حداکثر ارتفاع پله با توجه به رابطه‌های موجود در روش آزمون پله فرانسیس، تعیین و سپس پله موردنظر ساخته شده در اين مطالعه از ضریب همبستگی درون طبقه‌ای درون گروهی (Intraclass correlation coefficient) جهت بررسی پایایی پله، پس از انجام تست در 2 نوبت، با فاصله زمانی یک هفته‌ای بر روی یک گروه واحد استفاده شد همچنین جهت بررسی روایی پله نيز از ضریب همبستگی پیرسون استفاده شد. تمامی تحلیل‌ها در سطح معناداری 05/0 و توسط نرم‌افزار SPSS صورت گرفت. نتایج: نتایج آزمون سنجش پایایی با ضریب همبستگی ICC برابر 886/0، پایا بودن پله محقق ساخته را تأیید کرده است. همچنین روایی پله (737/0=r و 001/0>P) نيز مورد تأیید قرار گرفت. نتیجه‌گیری: نتايج مطالعه حاضر نشان داد که پله محقق ساخته جهت آزمون پله، از روايي و پايايي مناسبی برای اندازه‌گیری میزان VO2MAX برخوردار می‌باشد. این یافته‌ها نشان می‌دهد که می‌توان از این وسیله جهت سنجش میزان VO2MAX در افراد مختلف استفاده نمود.

Title: بررسی کمی و کیفیت متدولوژيك مقالات مجله علمی- پژوهشی دانشگاه علوم پزشکی شاهرود (دانش و تندرستی از سال 1386 - 1391)

http://knh.shmu.ac.ir/index.php/site/article/view/1360

مقدمه: یکی از شاخص‌های ارزیابی فعالیت‌هاي پژوهشی، تعداد و کیفیت مقالاتی است که در مجلات علمی- پژوهشی چاپ می‌شود. به‌منظور بررسی کمی و کیفی مقالات منتشر شده در مجله دانش و تندرستی، مطالعه حاضر طراحی‌ شده است. مواد و روش‌ها: پژوهش به‌روش توصیفی و نمونه‌گیری به طريق سرشماری انجام شد؛ و تعداد 23 شماره مجله مربوط‌به سال‌های 1386 تا پایان سال 1391 مورد بررسي قرار گرفت. یک چک ‌لیست محقق ساخته شامل شاخص‌های کمی و کیفی آماده شد: شاخص‌های کمی نظیر سال و شماره مجله، تعداد مقالات منتشر شده، فاصله زمانی تا چاپ مقاله تعداد كل نويسندگان، محل كار نويسنده اول، تعداد كلمات اختصاري در عنوان مقاله و تعداد کلمات کلیدی و شاخص‌های کیفی نظیر نوع طراحی و رعایت اصول مهم مقاله‌نویسی در بخش‌های مختلف شامل: عنوان، چکیده، مقدمه، مواد و روش‌ها، نتايج، بحث و منابع بود. از آمار توصیفی و روش آزمون مناسب برای نمایش داده‌ها و مقایسه آنها استفاده شد. نتايج: در مجموع تعداد 171 مقاله مورد بررسي قرار گرفت که میانگین تعداد مقالات به ازاء هر شماره 2±5/7 مقاله بود. کلیه مقالات حاصل کار تحقیقاتی اصیل (پژوهشی) بودند و مقاله مروری، تألیفی و یا نامه به سردبیر در هیچ شماره‌ای یافت نشد. میانگین زمان صرف شده از دریافت مقاله تا پذیرش آن 7/3±7/6 ماه است. تعداد 10 مقاله (6%) کار آزمایی بالینی بود. كاملترين و كم اشتباه‌ترين قسمت‌هاي مقالات به‌ترتيب منابع و عنوان مقالات بودند، در حالي‌كه قسمت بحث ضعيف‌ترين قسمت مقالات چاپ شده بود. نتیجه‌گیری: نتایج حاصل از بررسی فصلنامه علمی پژوهشی مجله دانش و تندرستی حاکی از آن است که در طول سال‌های انتشار به لحاظ کیفیت متدولوژيك پیشرفت داشته است و در مواردی نيز دچار کاستی‌ است. از این‌رو، مجموعه پیشنهادهایی جهت ارتقای بیشتر نشریه اريه می‌گردد.

Title: بررسي رابطه شادي و عملكرد كاركنان در دانشگاه علوم پزشكي شاهرود

http://knh.shmu.ac.ir/index.php/site/article/view/1282

مقدمه: شادي و نشاط به‌عنوان یکی از مهمترین نیازهای روانی بشر، به‌دلیل تأثیرات عمده‌ای که بر شکل‌گیری شخصیت آدمی و در یک کلام مجموعه زندگی انسان دارد، همیشه ذهن انسان را به خود مشغول کرده است و ايجاد سازمان شاد يكي از نيازهاي استراتژيك براي موفقيت در بلند مدت مي‌باشد. اين مطالعه با هدف بررسي رابطه شادي و عملكرد كاركنان در دانشگاه علوم پزشكي شاهرود در سال 1392 انجام گرديد. مواد و روش‌ها: در این پژوهش توصيفي همبستگي با استفاده از پرسشنامه شادي آکسفورد و پرسشنامه عملکرد هرسی و گلد اسمیت، 10 بعد اصلی شادي شامل: یادگیری سازمانی، خودگشودگی، مشارکت، عدالت سازمانی، مثبت‌اندیشی، ساختار منعطف، معناداربودن کار، علاقه به کار، امنیت و تعامل با همکاران و ارتباط آنها با عملكرد سازمانی افراد در 304 نفر مورد بررسی قرار گرفت. داده‌ها با استفاده از همبستگی پيرسون و رگرسیون چند متغيره تحليل گرديد. نتایج: نتايج پژوهش نشان داد مشارکت (87/0)، علاقه به کار (79/0)، یادگیری سازمانی (78/0) و عدالت (75/0) به‌ترتیب بیشترین همبستگی معني‌دار را با عملکرد سازمانی دارند و در نهایت شادي (73/0) همبستگي مستقيم و معناداري با عملکرد سازمانی دارد. نتيجه‌گيري: با عنايت به نتايج مطالعه لازم است مدیریت در جهت بهبود موارد یاد شده، به‌دنبال استراتژی‌های عملی و کاربردی باشد تا سطح شادي کارکنان را افزایش دهد و در نتیجه عملکرد کارکنان بهبود یابد.

Title: تأثیر هشت هفته تمرینات منتخب درمانی بر دامنه حرکتی و درد مبتلایان به سندرم گیرافتادگی شانه

http://knh.shmu.ac.ir/index.php/site/article/view/1209

مقدمه: سندروم گيرافتادگي شانه از علل بروز درد و محدوديت حركتی شانه محسوب مي‌شود و بهبود حرکتی عضلات کتف و روتیتور کاف اهمیت به‌سزایی در درمان این سندرم دارد. از این رو هدف پژوهش حاضر بررسی تأثیر 8 هفته تمرینات منتخب بر میزان دامنه حرکتی مبتلایان به سندرم گیرافتادگی شانه می‌باشد. مواد و روش‌ها: در این مطالعه، تعداد 24 مرد غیر ورزشکار با دامنه سنی 35 تا 55 سال داوطلبانه شرکت نمودند و به‌طور تصادفی در دو گروه 12 نفره تجربی و کنترل قرار گرفتند. سپس گروه تجربي در برنامه تمريني طراحي شده هشت هفته‌اي (سه جلسه در هفته) شرکت نمودند. در حالی که گروه کنترل فقط فعالیت‌هاي روزمره خود را انجام می‌دادند. حرکات مختلف شانه با استفاده از گونیامتر یونیورسال و نیز شدت درد با مقیاس بصری VAS قبل و بعد از مطالعه در دو گروه مورد ارزیابی قرار گرفت. از آمار توصیفی براي محاسبه میانگین‌ها و انحراف استاندارد داده‌ها استفاده شد و برای تعيين میزان تغییرات درون‌گروهی و بین‌گروهی، از آزمون تی همبسته و مستقل (05/0>P) با استفاده از نرم‌افزار SPSS استفاده شد. نتایج: نتایج تحقیق نشان داد که تمرینات منتخب در گروه تجربی به‌طور معنی‌داری باعث افزایش دامنه حرکتی حرکات شانه گردید و از میزان درد آنها نسبت‌به گروه کنترل کاسته شد. نتیجه‌گیری: نتایج به‌دست آمده حاکی از آن است که استفاده از حرکت درمانی می‌تواند مانند سایر روش‌های درمانی روشی مؤثر برای بهبود دامنه حرکتی و کاهش درد افراد مبتلا به سندرم گیرافتادگی شانه مورد توجه متخصصین قرار گيرد.

Title: بررسی تأثیر آموزش همتا بر خودکارآمدی ديابتي بيماران ديابتي نوع 2: يك مطالعه كارآزمايي باليني

http://knh.shmu.ac.ir/index.php/site/article/view/1359

مقدمه: توجه به ابعاد روانشناختی بیماران دیابتی، به‌ویژه خودکارآمدی آنها مهم بوده و می‌تواند در روند درمان این بیماران بسیار مفید باشد. آموزش به این بیماران ضروری بوده و آموزش همتا در تسهیل نمودن و ایجاد محیطی برای یادگیری تأثیر بسیار دارد. اين مطالعه با هدف تاثير آموزش همتا بر خودکارآمدی بيماران ديابتي انجام شد. مواد و روش‌ها: در این كارآزمايي باليني، 70 بیمار دیابتی نوع 2 که دارای ویژگی‌های شرکت در پژوهش بودند، در بیمارستان فیروزگر با نمونه‌گیری آسان وارد مطالعه شده و سپس تخصیص بیماران به دو گروه به‌روش تخصیص تصادفی بلوکی انجام شد. بیماران گروه مداخله توسط همتایاني كه آموزش ديده بودند، طی دو جلسه تحت آموزش قرار گرفته، اما بیماران گروه کنترل فقط آموزش روتین بخش را دریافت نمودند. ابزار گردآوری داده‌ها، پرسشنامه اطلاعات دموگرافیک و پرسشنامه خودکارآمدی در مديريت ديابت بود. خودكارآمدي بیماران قبل از آموزش و 2 ماه بعد از آموزش سنجیده شد. اطلاعات جمع‌آوری شده با کمک نرم‌افزار SPSS مورد تجزیه و تحلیل قرار گرفت. نتایج: میانگین خودكارآمدي دیابتی در هر دو گروه در روز بستری اختلاف آماری معناداری نداشتند (2/0=P). اما ميانگين خودكارآمدي دیابتی بيماران گروه مداخله در مقايسه با گروه كنترل در دو ماه بعد از آموزش تفاوت معني‌داري داشت (001/0P<). نتيجه‌گيري: با‌توجه به يافته‌هاي اين پژوهش به‌نظر می‌رسد، آموزش همتا براي بيماران دیابتی روشی مؤثر در جهت افزایش خودکارآمدی دیابتی بیماران می‌باشد. لذا توصيه مي‌شود جهت آموزش بيماران، از اين رويكرد آموزشي در بخش‌هاي بستری استفاده شود.
